# Coronasomnia in Employees without a Direct Contact with COVID-19 Infected Patients in Their Workplace

**DOI:** 10.3390/healthcare10071194

**Published:** 2022-06-26

**Authors:** Marina Ruxandra Oțelea, Corina Zugravu, Agripina Rașcu, Oana Cristina Arghir, Loredana Sabina Cornelia Manolescu, Dana Mates

**Affiliations:** 1 Clinical Department 5, Faculty of Medicine, University of Medicine and Pharmacy, Carol Davila, 020021 Bucharest, Romania; marina.otelea@umfcd.ro (M.R.O.); agripina.rascu@umfcd.ro (A.R.); 2 Department of Fundamentul Sciences, Faculty of Midwifery and Nursing, University of Medicine and Pharmacy, Carol Davila, 020021 Bucharest, Romania; 3 National Center for Monitoring the Community Risks, Institute of Public Health, 050463 Bucharest, Romania; dana.mates@insp.gov.ro; 4 Colentina Clinical Hospital, Department of Occupational Medicine, 21 Stefan cel Mare St, 020125 Bucharest, Romania; 5 Clinical Department 4, Faculty of Medicine, Ovidius University of Constanta, 1 University Alley, Campus, Corp B, 900470 Constanta, Romania; oana.arghir@univ-ovidius.ro; 6 Department of Microbiology, Parasitology and Virology, Faculty of Midwifery and Nursing, “Carol Davila” University of Medicine and Pharmacy, 050474 Bucharest, Romania; 7 Department of Virology, Institute of Virology “Stefan S. Nicolau”, 030304 Bucharest, Romania

**Keywords:** insomnia, coronasomnia, vulnerable health group, media consumption

## Abstract

Background: The aim of this analysis was to explore coronasomnia in second line workers. Methods: Data were collected via in an online questionnaire. Patients with new onset or aggravation of insomnia were defined as cases and those without insomnia as controls. Differences among groups were studied by nonparametric tests; the correlation among variables was assessed using regression, followed by Bonferroni adjustment. Results: There were 377 responders, grouped into 129 cases and 248 controls. Younger age (Odds Ratio = 0.97, *p* = 0.021), women (OR = 2.46, *p* = 0.016), workers belonging to a vulnerable group (OR = 2.36, *p* = 0.007), and those with previous history of insomnia (OR = 38.76, *p* = 0.00) were associated with coronasomnia. Increased home duties were directly related to insomnia (OR = versus home support which were indirectly associated). The constant preoccupation for SARS-CoV-2 media reports (OR = 3.6, *p* = 0.00009) and media consumption were components of the coronasomnia. In the adjusted models, the personal medical history, and the anxiety created by media alerts maintained their significance. Conclusion: Preventive measures to reduce the occurrence of insomnia in times of social stress in nonessential occupations should focus on health vulnerable groups, persons with previous history of insomnia and who develop anxiety from media.

## 1. Introduction

Coronasomnia defines a constellation of symptoms associated to the sleep impairment during the COVID-19 pandemic [1]. The main causes, as pointed out by research, are the high level of stress, changes in sleep routine and the uncertainties about the negative impact of disease [1,2,3,4]. The info media news alerts added specific elements of growing distress caused by the general uncertain perspective of infection and lockdown [2]. Linked to mass media information, a new term, “infodemic”, was defined as too much and contradictory information, leading to confusion and mistrust in public authorities during a disease outbreak and recognized as another major source of stress [3]. Challenges encountered in different occupations increased the prevalence of insomnia to 33–36% [4,5]. The majority of studies have focused on frontline workers exposed to COVID-19 infection that are involved in essential occupational activities, emphasizing the role of the workload, changes in procedures and schedules, combined with the fear about contamination, lack of efficient treatment and an overall decrease of efficacy with severe cases [6]. However, there were also other stressors for employees working with the general public [7], in education, welfare, and emergency services. Stress was present also in what was called the “non-essential activities”, for example, among people constrained to working from home during lockdown [8], or in confined spaces [9], who changed their occupational routine by teleworking, which creates technostress, a combination of work overload, invasion of privacy, role ambiguity and reduced social support [10].

Insomnia was a matter of concern for the Romanian population even before the pandemic [11]. Several studies have focused on first line workers [12], or on the general population [13], but a specific report focused on the second line workers is missing.

In a previous multidimensional approach, considering personal characteristics, occupational factors, and personal perception of risk, we investigated COVID-19 related insomnia among Romanian workers, revealing a higher, but not statistically significant prevalence of insomnia in healthcare workers compared to personnel from other occupational domains [14]. Therefore, we considered that a dedicated, in-depth analysis of second line workers, who were not in contact with COVID-19 patients at work, should be of interest, to a better understanding the factors that contribute to the development of insomnia.

## 2. Materials and Methods

### 2.1. Study Design

The study regarding insomnia had a cross-sectional design and the general findings were previously reported [14]. In this stage of the analysis, we focused only on second line workers having nonessential occupations regarding SARS-CoV-2 exposure. Even if these workers were not in the frontline and their job tasks were considered as safe related to the risk of SARS-CoV-2 infection, they were not necessarily at low risk for stress and/or mental health issues during the pandemic. 

A report of the International Labour Organization underlines the importance of different areas of intervention to prevent psychosocial risk during the pandemic which covers many other fields, such as job security, work-life balance, prevention of negative coping strategies [15]. 

The study group was selected in two steps: first, subjects who responded as having direct contact with COVID-19 patients during their work process were excluded. 

Cases were selected from second line workers with new onset or significant worsening of insomnia during the pandemic. Insomnia was recorded as a self-reported symptom, based on the positive answer to the question: “Did you experienced sleep difficulties (insomnia) initiated or significantly aggravated since the beginning of the pandemic”. The control group included persons without insomnia.

### 2.2. Instrumentation

In brief, the online survey was distributed through social media. The structure of the survey included four categories of possible contributory factors related to coronasomnia:

(a) The personal perception of the SARS-CoV-2 infection risk (fear of catching the disease at work, the infodemic, the anxiety perceived after watching the news).
(b) The personal vulnerability to infection (age, gender, chronic medical conditions, including previous insomnia episodes).
(c) Extra-professional strains: difficulty to perform household duties related to the pandemic, having a family member belonging to a vulnerable health group and the level of support provided by the family members.
(d) Occupational stressors (work overload, the changes in the working procedures or location, such as telework, and changes in the working schedule).

The vulnerable health item referred to the following pre-existing medical conditions: high blood pressure, obesity, diabetes, cancer, autoimmune diseases, chronic respiratory diseases, chronic kidney disease.

The psychological support provided by the organization was registered as well, as a possible protective organizational factor. 

The items of the survey were written in plain language. They were initially tested on a small group of people with different occupations and the feedback received was used to reword a few questions. The time for completion was considered acceptable and there was no skipping of answers due to confusion with understanding the meaning.

The questionnaire was distributed through invitations sent via email to the personal networks and social media accounts of the team members. Participation in the online survey was voluntary and anonymous. Prior to participation, the responders were informed of the objectives and each of them provided consent before responding to the questionnaire.

### 2.3. Statistical Analysis

After carrying out a descriptive analysis and the nonparametric tests (chi-square test) for qualitative variables and Mann–Whitney U for the quantitative ones, we conducted correlation tests between the items of the questionnaire assembled in the four categories of variables previously mentioned and insomnia. All data were processed in SPSS software (StatPlus:mac, a statistical analysis program for Mac OS Version 8, see AnalystSoft Inc., Walnut, CA, USA). For items grouped in each category of variables, Bonferroni adjustments were performed. A *p* value < 0.05 defined statistical significance for all statistical tests.

### 2.4. Ethical Approval

The study was peer-validated and approved by the Ethics Committee of the University of Medicine and Pharmacy Carol Davila, Bucharest, Romania (no 9985/11.05.2020); all the procedures of the study respect the ethical standards of the Helsinki Declaration. Informed consent was compulsory.

## 3. Results

From the total 769 persons who initially completed the online questionnaire, 377 responders declared no possible contact with COVID-19 patients during their work process. Of them, 129 (34.12%) were identified with insomnia and defined as cases and 248 without insomnia were considered controls. The prevalence of insomnia was higher in women from both cases and controls, but statistically non-significant (*n* = 108/129; 83.4% vs. *n* = 187/248; 75.4%; χ^2^ = 3.44, *p* = 0.06) (Table 1). 

The average age was similar in cases (41.28 years, with standard deviation (SD) = 11.16) and controls (42.51, with SD = 11.07). The tenure in the current job was, on average 11.46 years (SD = 11) compared to 12.4 (SD = 10.13) in the control group. There was no statistically significant difference either in age, or in tenure between the two groups.

The main characteristics of cases and controls, revealed by the online survey, are presented in Table 1. The prevalence of previous insomnia was 17.24% in the whole sample, whereas in cases it was 34.12% (χ^2^ = 111.59, *p* < 0.0001). From the total 312 persons who were good sleepers before the pandemic, 70 (22.44%) developed insomnia. 

The most significant factor associated with insomnia was the personal concerns about the risk of SARS-CoV-2 infection. Fear of going to work, infodemia, anxiety effects of watching media, and previous personal history of insomnia were strongly related to coronasomnia (*p* < 0.001). Four supplementary questions analysed the fear of going to work and these identified the potential causes as: insufficient knowledge about the transmission of the SARS-CoV-2 infection, lack of efficacy of protective measures, lack of an efficient treatment, and the lack of instruction about the personal safety. All items were directly related to the fear of going to work. The regression analysis had as the dependent variable insomnia, and all these causes, as independent variables, showed that only the lack of an efficient treatment remained significant (Odds Ratio = 2.79, CI = 1.28–6.07, *p* = 0.009).

Percentages were calculated from the total number of responders in each group category. Missing data (subjects choosing the option “I prefer not to answer” or “not applicable”) were not included, either in the total, or in the percentage calculation. 

In a multivariate analysis, all personal factors correlated with insomnia were evaluated as significant contributors to impairment caused by the COVID-19 pandemic (Table 2). After the Bonferroni correction, belonging to a vulnerable health group and a personal history of insomnia were the only variables with significant association.

The infodemic and anxiety, perceived after following the media and alert news, were identified as the main components of concerns regarding the risk of SARS-CoV-2 infection, which maintained their statistical significance in the multivariate regression model (Table 3). Applying the Bonferroni correction, only anxiety related to the media news had a significant influence on insomnia.

Concerning the extra-professional strains induced by the pandemic, the main influencer seemed to be the difficulty to solve the household duties in the context of the restrictions imposed by the authorities during the pandemic (Table 4).

## 4. Discussion

Studies conducted before the pandemic showed that the prevalence of insomnia tended to remain stable in a population [16], with variable figures, ranging from 3.9–30% [17,18]. Before the pandemic, an internet survey using as the instrument of data collection the Basic Nordic Sleep Questionnaire, found that one fifth of the Romanian population suffered from insomnia [11]. In that study, the prevalence was not influenced by the working schedule. 

But the pandemic changed the picture of the sleep disorders [19]. Clinically significant insomnia in the general population rose to 20–32% [20,21]. In a group of second line workers not exposed to a direct contact with COVID-19 patients, we found an even higher prevalence of insomnia. This is in accordance with the boost in mental health issues. In a Romanian national survey, more than a quarter of the population (26.5%) perceived a high level of stress (higher than 8, on a scale of 1 to 10) during the isolation and quarantine caused by COVID-19 pandemic [22]. Anxiety was reported as high by 60% of the Romanian respondents in a study carried out during the pandemic, in a mostly urban sample [23].

Moderate to severe levels of depressive symptoms were experienced by one in five, and moderate to severe levels by one in four Romanians [24]. More significant, in this study, the symptoms of anxiety and depression were maintained during the COVID-19 pandemic at approximatively the same levels. Stressful situations or events reduced the sleeping duration and impaired the architecture of the sleep; a longer sleep latency, an increase in the REM density and increased number of awakenings were the major modifications induced by stress [13]. 

Our data are slightly higher than those calculated by Krishnamoorthy Y. et al. in a meta-analysis of 50 studies conducted during the pandemic [25] and certainly varies with the collection method. An explanation could be that we considered as cases not only the ones with insomnia onset during the COVID-19 pandemic, but also the ones which had been significantly aggravated by the pandemic alerts context, and the symptoms presence was sufficient for including them in the cases group. 

Patients with chronic diseases have a significantly increased chance of insomnia occurrence [26,27,28]. There are many factors contributing to this result: severe pain, depression, and anxiety, to which the pandemic added specific supplementary elements, such as isolation, the difficulty for getting the proper medical assistance in adequate time and the fear of getting severe complications, death, or long COVID-19. Impaired access to care has been reported in many countries, and Romania does not make an exception for many medical or surgical specialties [29,30,31].

It is not only the already established comorbidities that aggravate the evolution and prognosis of COVID-19 (diabetes, heart disease, chronic renal disease, immunodepression) [32,33], but also respiratory [34] and neurological diseases [35,36], as well as kidney transplant [37], were conditions associated with an increased risk of insomnia. Our results confirm these findings, as the responders in our survey with at least one chronic medical condition had an increased chance of developing insomnia.

Belonging to a vulnerable group may result in an individual perceiving a higher workload [38]. We did not notice this perceived workload factor in our study sample, probably because we measured the relative increase and not the absolute perception of the workload. Another explanation could be the fact that vulnerable people would have more benefit from doing their work at home.

All items summarized in the category of concern and materialization of the risk of SARS-CoV-2 infection were significantly related to insomnia in the univariate analysis. The fear of going to work was a surprising result, as this category of second line employees were not in contact with COVID-19 during their working hours. Fear is commonly perceived as dysfunctional, but it was shown to also have a functional role in the pandemic, mainly by developing a proactive attitude towards prevention [39]. In our study, the fear of infection caused by SARS-CoV-2, was probably more dysfunctional, as, in the multivariate regression, insomnia was significantly related only to the efficacy of treatment. The immediate threat, represented by infection, triggers a red flag fight response, which decreases the efficacy of work, by impairing problem solving processes, the memory and constructive thoughts, social relatedness, and autonomy [40]. A list of COVID-19 related fears, which has been validated in a study in Romanian language [41], included among its seven items, one related to sleep disturbance (“I cannot sleep because I am worrying about getting COVID-19”). The validation study not only confirmed the internal validity of the questionnaire, but also its concurrent validity, namely the correlations with stress, depression, resilience, and happiness. The inclusion of the sleep related item is perfectly justified, as insomnia itself leads to emotional dysregulation [42], which, in turn, exacerbates the fear. From the cross-sectional design of our study, we cannot conclude whether fear is the main cause of insomnia, or the causality goes in the opposite direction. 

The infodemic and the anxiety induced by the COVID-19 news media both maintained their statistical significance in the adjusted regression of the concerns about the high risk of imminent infection. This was acknowledged from the early days of the pandemic and even for healthcare professionals [43]. The healthcare personnel who were affected by news, suffered more from insomnia, and had higher levels of stress and anxiety. More than 3 h of television viewing and 4 h of internet computer usage or mobile phone usage was associated with significantly greater odds of having poor sleep quality [44], even before the pandemic. The number of hours of television watching per day increased during COVID-19, to the detriment of the number of hours of physical activity [45]. 

Compared to the data for the total sample of responders, which was presented in a previous communication [14], this current analysis that targeted the second line workers underlines some distinct features, mainly the major influence of the non-occupational stressors and the lack of statistical association with the occupational ones (work overload, the changes in the working procedures or location, and changes in the working schedule).

Occupational stressors were not related to insomnia in this particular group. This was expected, as working with COVID-19 patients was a direct influencer of insomnia in the previous full-sample analysis and is concordant with other research studies. For example, the same distinction was found in healthcare workers from COVID-19 and non-COVID-19 hospitals [46]. Personal resilience is a well-characterized protector factor for insomnia and distress [47] and some organizations that have provided training to increasing resilience had positive effects on their employees. In our study, psychological support was provided in less than a quarter of the organizations and most of the insomniacs did not have access to this type of support. It has been shown that good communication, organizational and social support, proper protection, and positive work attitudes [48] can decrease the level of stress. Even if the perception of risk depends on personal traits, such as neuroticism and conscientiousness [49], resilience training, inside the organization, has been proven beneficial for coping with the challenges imposed by the COVID-19 pandemic [50]. In fact, like our findings, concerns about COVID-19 were more important than the actual risk of infection at work in predicting insomnia [51,52,53,54,55,56].

### Strengths and Weakness

There is certainly a limitation due to the cross-sectional design and the subjective definition of insomnia.

There are no standard definitions for insomnia or sleep problems in epidemiological research [57], making the comparison between studies difficult because of the different definitions of insomnia. Broadly, insomnia covers the long sleep latency, the short duration and/or the poor quality of sleep [19] and the questions used in this study were general enough to cover all these items. We selected this approach, even if it does not give a measure of severity, because several systematic reviews have concluded that an extensive validation of the questionnaire evaluating insomnia is rather limited [58].

Another limitation is the number of participants, which did not allow clustering analysis. For example, the wide distribution of occupations resulted in some occupational groups being underrepresented compared to others. Therefore, we cannot extrapolate the lack of association with workload to all second line workers and further studies covering large enough samples of occupational groups should be performed to clarify this relation.

A strength of this study is the comprehensive investigation of occupational and non-occupational risk factors for insomnia in the current COVID-19 pandemic and the thorough full selection of the nonessential workers.

## 5. Conclusions

Our results support the conclusion that non-occupational risk factors influenced insomnia in persons who did not have direct contact during working hours with COVID-19 patients. 

Chronic medical conditions and, particularly, a personal previous history of insomnia make these people more vulnerable. The infodemic increases anxiety and finding appropriate ways to address the concerns and questions of the general population, together with building resilience to misinformation, are important issues to be addressed now, to mitigate the mental health impacts of future social, economic or medical crisis.

## Figures and Tables

**Table 1:** Description of the main characteristics of the groups.

Variable	Cases No (% of Cases)	Controls No (% of Controls)	Total No (%)
Women	108 (83.72%)	187 (75.4%)	295 (78.25%)
Men	21 (16.28%)	61 (24.6%)	82 (21.75%)
Belonging to a vulnerable health group (no/%) *	43 (33.86%)	55 (22.18%)	98 (26.13%)
Previous history of insomnia **	59 (45.74%)	6 (2.42%)	65 (17.24%)
Concern and materialization of the risk of infection			
Having a colleague or a close relation diagnosed with COVID-19 *	25 (20%)	28 (12.02%)	53 (14.8%)
Contact or diagnose with COVID-19	6 (4.76%)	8 (3.25%)	14 (3.76%)
Fear of going to work **	56 (61.53%)	66 (35.49%)	122 (44.04%)
The infodemic **	109 (87.2%)	162 (68.64%)	271 (75.07%)
The anxiety effects of the media news **	86 (66.67%)	71 (28.63%)	157 (41.64%)
Extra-professional strains			
Difficulties in solving the household tasks related to the pandemic *	65 (50.39%)	93 (37.5%)	158 (41.91%)
Family member belonging to a vulnerable health group	68 (52.71%)	125 (50.4%)	193 (51.19%)
Adequate support provided by the family members *	119 (92.24%)	241 (97.18%)	360 (95.49%)
Occupational stressors			
Increased work overload	100 (77.52%)	172 (69.35%)	272 (72.14%)
Changes in the working location	25 (19.38%)	32 (12.9%)	57 (15.12%)
Changes of working schedules	101 (78.29%)	178 (71.77%)	279 (74.01%)
New working procedures	24 (27.59%)	48 (29.27%)	72 (28.68%)
Psychological support provided by the organisation	20 (18.01%)	52 (23.85%)	72 (21.88%)

* *p* < 0.05; ** *p* < 0.001.

**Table 2:** Personal contributory factors to coronasomnia.

Variable	Odds Ratio	LCL	UCL	*p*
Age	0.97	0.94	0.99	0.021
Gender	2.46	1.18	5.1	0.016
Belonging to a vulnerable health group	2.36	1.26	4.43	0.007
Personal history of insomnia	38.76	15.59	96.4	0.00

LCL = low confidence limit; UCL = upper confidence limit.

**Table 3:** Factors of concern about the COVID-19 infection.

Variable	Odds Ratio	LCL	UCL	*p*
Having a colleague or a close relation diagnosed with COVID-9	1.5	0.69	3.24	0.30
Contact or diagnose with COVID-19	1.08	0.24	4.92	0.92
Fear of going to work	1.12	0.94	1.33	0.20
The infodemic	2.6	1.07	6.36	0.04
The anxiety effects of the media news	3.6	1.9	6.86	0.00009

LCL = low confidence limit; UCL = upper confidence limit.

**Table 4:** Extra-professional burden during COVID-19 pandemic.

Variable	Odds Ratio	LCL	UCL	*p*
Difficulties in solving the household tasks related to the pandemic	1.57	1.01	2.44	0.046
Family member belonging to a vulnerable health group	1.18	0.74	1.87	0.48
Adequate support provided by the family members	0.39	0.15	1.09	0.07

LCL = low confidence limit; UCL = upper confidence limit.

## Data Availability

Not applicable.
